# Interactions of clinical relevance associated with concurrent administration of prescription drug and food or medicinal plants: a systematic review protocol

**DOI:** 10.1186/s13643-019-1259-2

**Published:** 2020-01-06

**Authors:** Adriana Orellana-Paucar, Daniela Vintimilla-Rojas

**Affiliations:** Carrera de Nutrición y Dietética, Facultad de Ciencias Médicas, Universidad de Cuenca, Cuenca, Ecuador

**Keywords:** Concurrent administration, Pharmacological interaction, Food-drug interaction, herb-drug interaction, Safety, Human

## Abstract

**Background:**

An inadequate combination of prescription drugs with food or medicinal plants could cause adverse effects in patients or produce negative therapeutic results. Therefore, this generic systematic review protocol aims to identify and synthesize the literature on clinical characteristics and safety issues of these types of pharmacological interactions occurring in children, adolescents, adults, pregnant/lactating women, and older adults.

**Methods/design:**

This generic protocol follows the stated guidelines from the Preferred Reporting Items for Systematic Reviews and Meta-Analyses Protocols (PRISMA-P) statement. A literature search will be performed in PubMed, Scopus, and Virtual Health Library (VHL) electronic databases from 1960 till present for studies reporting clinical characteristics and safety issues associated with pharmacological interactions occurring between prescription drugs and food or medicinal plants in participants from birth-age to ≥ 65-year-old, including pregnant/lactating women. Lateral searching will be carried out in PubMed via related citation. Two reviewers will carry out an independent evaluation of eligible studies as well as the corresponding data extraction of the selected ones. Subsequently, the methodological quality evaluation of the selected articles will be completed using the corresponding Joanna Briggs Institute Checklists. Moreover, the quality of evidence will be graded according to the criteria of the Grading of Recommendations, Assessment, Development and Evaluation (GRADE) Working Group. Quantitative research in humans comprising clinical trials and clinical, comparative and, observational studies will be included. The main outcomes of this protocol involve reported potential food-drug and herb-drug interactions, associated safety issues, and adverse reactions along with the generic name of the prescribed drug and the scientific name of the food and medicinal plants involved in these types of pharmacological interactions. Finally, findings extracted from the selected studies will be summarized in a narrative synthesis.

**Discussion:**

This generic systematic review protocol seeks to synthesize and critically evaluate current knowledge besides to identify any comprehension gaps in the concurrent administration of prescription drugs with food and herbs. By achieving a better understanding of this topic, this information will allow healthcare professionals to develop useful strategies to recognize, manage, and prevent these types of pharmacological interactions at different age stages, including pregnant/lactating women.

**Systematic review registration:**

PROSPERO CRD42018117308

## Background

Drug-drug interactions are well known among healthcare professionals. Consequently, they are often avoided in medical prescriptions or quickly treated when clinically recognized [1]. In contrast, information on food-drug and herb-drug interactions is not enough widespread and therefore, it turns to be less straightforward to identify and to treat opportunely [2–5].

Worldwide, food-drug and herb-drug interactions are a major health problem due to the risk of potential adverse reactions [6, 7]. Pharmacokinetic and pharmacodynamic variations derived from this type of interactions can produce toxicity or sub-therapeutic results associated with undesirable clinical consequences [8–12] such as the inhibiting action of tea (flavonoids), coffee (polyphenols), or dairy products (calcium) on iron supplements absorption [13–15]. Similarly, coumarin, a constituent of chamomile (*Matricaria chamomilla*), interacts with warfarin thus increasing the risk of hemorrhage [16, 17]. Likewise, concurrent administration of fluoroquinolones (ciprofloxacin) and dairy products or other foods fortified with calcium can decrease the drug’s bioavailability and could result in resistance to this class of antibiotics [18, 19].

Regarding this emerging problem of Public Health, the World Health Organization (WHO) is currently promoting specific strategies for the prevention of these kinds of pharmacological interactions through the establishment of public policies that support the diffusion of knowledge on the subject [20]. Likewise, in 2004, Germany published a reference guide for the evaluation of potential pharmacokinetic interactions between prescription drugs and herbal products. The 2012 “Guideline on the investigation of drug interactions” published by the European Medicines Agency contains chapters committed to food (Chapter 5) and herbal products (Chapter 6), thus highlighting the actual need to investigate their potential interactions with prescription drugs [21]. Correspondingly, the Regulatory Agencies of the European Union, the USA and Canada have established the obligation to mention on the label of herbal products, their possible interactions with prescription drugs, in case of confirmed evidence [22–25]. In reference to food-drug interactions, although scientific knowledge is available, there is still little awareness of the necessity to prevent them through government policies [3].

In Ecuador, the National Agency for Health Regulation, Control and Vigilance (Agencia Nacional de Regulación, Control y Vigilancia, ARCSA) through the National Pharmacovigilance Center carries out monitoring of adverse reactions caused by drug-drug interactions without considering potential interactions of prescription drugs with nutrients or with medicinal plants despite the fact that the “medicines, supplies, knowledge and use of medicinal plants” issue was considered as one of the research priorities of the Ministry of Public Health of Ecuador during the period 2013-2017 [26, 27].

The majority of drug-drug interactions are known and preventable, nevertheless little is known about pharmacological interactions between drugs and food or medicinal plants. Interactions between drugs and food or medicinal plants are not easily identifiable. However, its potential frequency is greater since food and herbal products are frequently associated with drug administration [28]. Although there is available information about this issue, it is not compiled in a review and therefore it is not easily accessible and comprehensible. A systematic review on the subject will favor prevention of adverse reactions caused by pharmacological interactions [29], prevention of complications of the disease under treatment due to toxicity, sub-therapeutic doses or unwanted clinical consequences [30–34]; reduction of hospitalization costs related to pharmacological interactions [35–37] and the availability of a reliable and updated body of knowledge regarding potential pharmacological interactions of food-drug and herb-drug types. Thus, a systematic review in this topic would indirectly support the obtaining of a satisfactory result with prescribed pharmacological therapy. Likewise, it would probably encourage the development of a culture of rational and responsible use of medicines with a focus on the appropriate combination with nutrients and medicinal plants among patients, nutritionists, pharmacists, and medical doctors.

## Aims

This systematic review protocol aims to identify, synthesize, and critically evaluate the scientific literature on clinical characteristics and safety issues associated with pharmacological interactions between prescription drugs and food or medicinal plants in children, adolescents, adults, pregnant/lactating women, and older adults.

### Review questions

The questions to be answered through the application of this review protocol will be:

- What clinical characteristics allow identifying and therefore, preventing pharmacological interactions occurring between a prescription drug and food or medicinal plant?
- What safety issues are related to concurrent administration of a prescription drug and food or medicinal plant?

## Methods/design

### Protocol and registration

The methodology to perform this systematic review was developed according to recommendations from the Preferred Reporting Items for Systematic Reviews and Meta-Analyses Protocols (PRISMA-P) statement [38]. The corresponding PRISMA-P Checklist is available in Additional file 1.

This protocol is currently registered in the International Prospective Register of Systematic Reviews (PROSPERO): CRD42018117308.

### Eligibility criteria

Research studies in humans assessing pharmacological interactions between a prescription drug and food or medicinal plant concurrently administered in male or female participants from birth-age to ≥ 65-year-old will be included. Inclusion criteria involve pregnant and lactating women as well.

### Type of studies

The following study designs will be included to answer review questions on clinical characteristics and safety issues associated with pharmacological interactions occurring between prescription drugs and food or medicinal plants:

- Quantitative research articles which may include clinical trials and, clinical, comparative and observational studies;
- Case reports of adverse effects derived from pharmacological interaction occurring between prescription drugs and food or medicinal plants.

Studies on the combination of products such as those containing herbal and non-herbal ingredients (e.g., vitamins, minerals) will be excluded as well as secondary and tertiary sources of information such as editorials, letters, and reviews.

Main outcomes of interest include potential food-drug or herb-drug interactions reported, associated safety issues and adverse reactions described as well as the generic name of the prescribed drug and the scientific name of the food or medicinal plant involved in these types of pharmacological interactions.

### Literature search

Literature search will be carried out in PubMed, Scopus, and Virtual Health Library (VHL) electronic databases from January 1960 till present using Medical Subject Headings (MeSH): “food-drug interaction,” “plant-drug interaction”/“herbal drug interaction,” the generic name of the prescription drug of interest (e.g., phenytoin) and the specific disease name whose treatment corresponds to the prescription drug of interest (e.g., epilepsy). The language of publication will be restricted to English and Spanish.

The search strategy was developed according to Patient/Problem, Intervention, Comparison group and Outcome (PICO) recommendations. As an example, the search strategy in PubMed for phenytoin and epilepsy is available in Additional file 2.

Deduplication will be applied in order to remove repeated studies. Subsequently, a lateral searching using the related citation function in PubMed in the selected studies will be carried out to avoid involuntary omission of relevant articles.

### Study selection process

Search results from all databases will be downloaded into Zotero® and merged to delete repeated articles. Two reviewers will individually check titles and abstracts of all obtained studies looking for potential inclusion. In case of uncertainty about relevance for an article, this will be reserved and its full text will be completely reviewed. Full text of all articles that may meet the eligibility criteria will be downloaded into Zotero®.

Applying a previously designed eligibility checklist matrix, two reviewers will independently evaluate the full text of all obtained articles against the eligibility criteria and will be correspondingly recorded into this matrix. Studies that do not entirely fulfill the determined inclusion criteria will be excluded and the correspondent reason(s) registered. Any disagreement on eligibility will be resolved by rationale argumentation between the two reviewers. A third reviewer will be consulted in case no consensus could be achieved.

### Data extraction

In a data extraction matrix, two reviewers will independently register the information of interest obtained from included studies. The two reviewers will newly examine the corresponding article(s) for data confirmation in case of disagreement on any record(s).

Following is described the essential data to be obtained:

- Publication details: author(s), publication year, language, title, journal, and country where the study was conducted and funding information;
- Study design: type of study (case report, clinical study, clinical trial, case-control study, cohort study, observational study), recruitment method(s) and data collection method(s);
- Study objectives: aims, outcomes, and measurement of outcomes;
- Participants: number of participants, demographic, and socio-economic characteristics, diagnosed disease (e.g., epilepsy), age group (child [birth to 18 years], adult [19 to 65 years], older adult [more than 65 years] and pregnant/lactating women);
- Prescription drugs: generic name of the prescription drug (e.g., phenytoin) and administered daily dose;
- Pharmacological interaction: type of pharmacological interaction reported for the prescription drug of interest (food-drug interaction or herb-drug interaction);
- Food: type of food (e.g., fruits, vegetables, dairy products, among others), specific name (e.g., cow’s milk, beef) or scientific name (in case of vegetables and fruits) involved in pharmacological interaction with prescription drug(s) and reported consumed amount;
- Herbs: scientific name of the plant, type of herbal preparation (e.g., medicinal plant infusion, herbal products, among others) and reported consumed amount or reported administered dose of active principles;
- Safety issues: adverse reactions reported; and,
- Study limitations: response bias, selection bias, among others.

### Methodological quality assessment

The Joanna Briggs Institute (JBI) Critical Appraisal Tools will be used to assess the methodological quality of each selected article [39]. The JBI Tools will be applied according to the type of study (e.g., for clinical studies and clinical trials, the Checklist for Quasi-Experimental Studies or the Checklist for Randomized Controlled Trials, according to the case. Likewise, the Checklists for Case Report Studies, for Case-Control Studies, for Cohort Studies, and, for Prevalence Studies are also available).

Two reviewers using the corresponding JBI Tools will independently evaluate the methodological quality of each eligible article meeting the inclusion criteria of this review protocol.

### Quality of evidence evaluation

The quality of evidence will be independently rated by two reviewers according to the criteria of the Grading of Recommendations, Assessment, Development, and Evaluation (GRADE) Working Group [40].

### Data synthesis

Data extracted from eligible studies will be synthesized using a textual narrative approach [41]. The synthesis will aim to describe the existing body of literature as well as to identify the scope of what has been studied, the strength of available evidence, and the gaps that currently need to be filled.

Findings will be summarized in a narrative interpretation focused on the review questions to provide a better understanding of clinical characteristics and safety issues associated with pharmacological interactions occurring between a prescription drug of interest and food or medicinal plants.

Results of the literature search and study selection process will be depicted in a flowchart [42]. Extracted relevant data will be summarized and shown in a table. Furthermore, a complete description of the research characteristics including details of study types, population and outcomes will be also presented. If necessary, data will be depicted in tables and graphs. Limitations of selected studies will be deeply discussed as well as their inferences on the findings obtained from the application of this protocol review. Gaps in knowledge will be identified and suggestions for future research on the field will be also provided.

## Discussion

Inappropriate combination of prescription drug (e.g., phenytoin) with food or herbs could lead to toxic or sub-therapeutic effects affecting the pharmacological treatment of a specific disease (e.g., epilepsy) or even to increased related hospitalization costs [29, 33–35, 37].

Although it is possible to find systematic reviews collecting information regarding a sort of drugs used for a specific disease solely focused on drug-food interactions or exclusively on drug-herb interactions, to date, it is unusual to find systematic reviews on clinical characteristics and safety issues related to concurrent drug administration with food and medicinal plants compiled as a whole in just one database for further dissemination.

Since this generic protocol can be applied not only for epilepsy, as mentioned in the examples, but for any disease treated with drug therapy, the application of this review protocol will generate a series of reviews that will synthesize and critically evaluate current knowledge. In addition, they will identify any gaps in knowledge thus providing important baseline data for future intervention research in this field.

The reviews derived from this protocol will summarize revised scientific information thus favoring the development of useful healthcare strategies to identify, manage and prevent these types of potential pharmacological interactions based on a better understanding of them.

### Dissemination

Findings obtained from the application of this generic systematic review protocol will constitute a baseline database related to food-drug and herb-drug interactions. Likewise, they will be summarized following PRISMA reporting standards and submitted to a peer-reviewed journal with a focus on drug safety as well as presented at linked scientific events.

## Supplementary information

**Additional file 1.** Preferred Reporting Items for Systematic Review and Meta-Analysis Protocols (PRISMA-P) checklist showing information regarding this systematic review protocol

**Additional file 2.** Search strategy used in this protocol for PubMed with detailed information regarding keywords, Boolean terms, Medical Subject Headings (MeSH), search terms and combination of search terms

## Abbreviations

AO-P: Adriana Orellana-Paucar
ARCSA: Agencia Nacional de Regulación, Control y Vigilancia (National Agency for Health Regulation, Control and Vigilance, Ecuador)
DV-R: Daniela Vintimilla-Rojas
MeSH: Medical Subject Headings
PICO: Patient/Problem, Intervention, Comparison group and Outcome
PRISMA: Preferred Reporting Items for Systematic Reviews and Meta-Analysis
PRISMA-P: Preferred Reporting Items for Systematic Review and Meta-Analysis Protocols
PROSPERO: International Prospective Register of Systematic Reviews
VHL: Virtual Health Library
WHO: World Health Organization
