# Supplementary material for: Interactions of clinical relevance associated with concurrent administration of prescription drug and food or medicinal plants: a systematic review protocol
Source: Syst Rev. 2020 Jan 6;9:1. doi: 10.1186/s13643-019-1259-2 (PMC6945643; doi:10.1186/s13643-019-1259-2)
Supplement: Supplementary file 2 — Additional file 2. Search strategy used in this protocol for PubMed with detailed information regarding keywords, Boolean terms, Medical Subject Headings (MeSH), search terms and combination of search terms [file 13643_2019_1259_MOESM2_ESM.docx]

**Search strategy for phenytoin and epilepsy**

**PubMed**

| 1 | phenytoin [Mesh] |
| --- | --- |
| 2 | epilepsy [Mesh] OR epilep* OR seizure OR aura |
| 3 | "food-drug interaction" [Mesh] OR “food drug interaction” OR “food-drug” OR “drug-food” OR “food interaction” |
| 4 | “herb-drug interaction"[Mesh] OR “herb drug interaction” OR “herb-drug” OR “drug-herb” OR “plant-drug interaction" OR “plant drug interaction” OR “plant-drug” OR “drug-plant” OR "herbal drug interaction" OR “herbal-drug interaction” OR “herbal-drug” OR “drug-herbal” |
|  |  |
|  |  |
